# Gypenoside L Inhibits Proliferation of Liver and Esophageal Cancer Cells by Inducing Senescence

**DOI:** 10.3390/molecules24061054

**Published:** 2019-03-18

**Authors:** Jingxin Ma, Xiaopeng Hu, Chenghui Liao, Haitao Xiao, Qinchang Zhu, Ying Li, Zhigang Liu, Anjin Tao, Zhendan He, Chenshu Xu, Kai Zheng

**Affiliations:** 1 School of Pharmaceutical Sciences, Shenzhen Key Laboratory of Novel Natural Health Care Products, Engineering Laboratory of Shenzhen Natural small molecule Innovative Drugs, Health Science Center, Shenzhen University, Shenzhen 518060, China; 18565811720@163.com (J.M.); hu54398@2008.sina.com (X.H.); liao.chenghui66@gmail.com (C.L.); Xhaitao@szu.edu.cn (H.X.); Zhuqc@szu.edu.cn (Q.Z.); li.ying@szu.edu.cn (Y.L.); lzg@szu.edu.cn (Z.L.); hezhendan@szu.edu.cn (Z.H.); 2 Hybio Pharmaceutical Co., Ltd. Shenzhen 518057, China; taoanjin@hybio.com.cn; 3 Guangdong Key Laboratory for Genome Stability & Human Disease Prevention, Health Science Center, Shenzhen University, Shenzhen 518060, China

**Keywords:** gypenoside L, senescence, NF-κB signaling, MAPK, cell cycle arrest, chemoresistance

## Abstract

Senescence is an irreversible state of cell cycle arrest that can be triggered by multiple stimuli, such as oxygen reactive species and DNA damage. Growing evidence has proven that senescence is a tumor-suppressive approach in cancer treatment. Therefore, developing novel agents that modulate senescence may be an alternative strategy against cancer. In our study, we investigated the inhibitory effect of gypenoside L (Gyp-L), a saponin isolated from Gynostemma pentaphyllum, on cancer cell growth. We found that Gyp-L increased the SA-β-galactosidase activity, promoted the production of senescence-associated secretory cytokines, and inhibited cell proliferation of human liver and esophageal cancer cells. Moreover, Gyp-L caused cell cycle arrest at S phase, and activated senescence-related cell cycle inhibitor proteins (p21 and p27) and their upstream regulators. In addition, Gyp-L activated p38 and ERK MAPK pathways and NF-κB pathway to induce senescence. Consistently, adding chemical inhibitors efficiently counteracted the Gyp-L-mediated senescence, growth inhibition, and cell cycle arrest in cancer cells. Furthermore, treatment with Gyp-L, enhanced the cytotoxicity of clinic therapeutic drugs, including 5-fluorouracil and cisplatin, on cancer cells. Overall, these results indicate that Gyp-L inhibits proliferation of cancer cells by inducing senescence and renders cancer cells more sensitive to chemotherapy.

## 1. Introduction

The current therapeutic strategies against cancer include chemotherapy, radiotherapy, surgery or combinations of them. Treatment of cancer by DNA-damaging chemotherapy is based on the principle that the genomic instability of cancers has a greater propensity to accumulate DNA damage. Genotoxic drugs, such as alkylating agents, mainly cause DNA fracture and interfere with DNA replication to inhibit tumor cell division, which usually trigger DNA damage repair response (DDR) [1,2]. The DDR is a complicated signaling network integrating cell cycle checkpoints, DNA repair and damage tolerance pathways to allow orderly completion of genome duplication and cell cycle progression. Therefore, cancer cells have evolved to usurp various strategies to promote cell cycle progression, to evade cell death and to develop chemoresistance [3,4].

A growing amount of evidence has proved that senescence is a critical tumor-suppressive approach in cancer treatment and prevention [5,6,7]. Senescence is a stress-activated genetic program that permanently prevents cells from further proliferation and is an irreversible state of cell cycle arrest. Senescent cells are characterized by a flat and enlarged morphology, elevated senescence-associated β-galactosidase (SA-β-gal) activity and the activation of several cell cycle arrest regulators, mainly p53-p21 and p16-Rb signaling pathways [8]. Senescence also leads to secretion of diverse cytokines, chemokines, various growth factors and proteases/inhibitors, which constitute the senescence-associated secretory phenotype (SASP) [9]. Multiple therapeutic treatments such as chemotherapeutic drugs, radiation, or hypoxia can induce senescence in cancer cells [5,6,7]. Recent evidence proved that several intracellular signals, such as MAPK, ROS, autophagy, and NF-κB pathway, are relevant for senescence induction [8,10,11,12]. These pathways are considered as a crucial tumor-suppressor mechanism for strengthen therapeutic drugs. Therefore, specific compound that induces senescence of cancer cells is promising for cancer treatment.

Natural products are important resources for anticancer drugs [13,14]. Gynostemma pentaphyllum is widely accepted as the complementary and alternative medicine in cancer therapy. G. pentaphyllum is well-established for its powerful adaptogenic and antioxidant effects and is traditionally used to maintain the normal function of liver, cardiovascular and digestive systems [15,16,17,18,19]. Previously we have identified and demonstrated that gypenoside L (Gyp-L), a saponin isolated from G. pentaphyllum, induced lysosome dysfunction and inhibited autophagic flux in cancer cells [20,21,22]. In the present study, we further revealed that Gyp-L induces senescence to inhibit the growth of human liver and esophageal cancer cells. Moreover, the underlying molecular mechanisms were investigated.

## 2. Results

### 2.1. Gyp-L Induces Senescence in Cancer Cells

**Figure 1:**
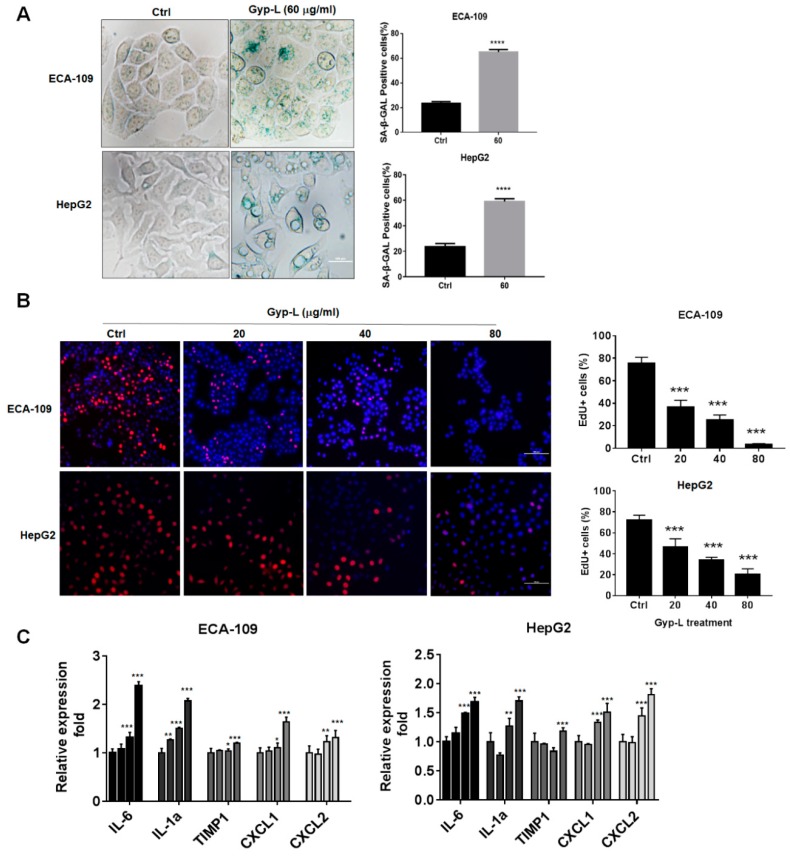
Gyp-L induced senescence in cancer cells. (**A**) Gyp-L increased SA-β-gal activity. Hepatic cancer cell HepG2 and esophageal cancer cell ECA-109 were treated with Gyp-L for 24 h and stained with SA-β-gal activity. The ratio of SA-β-gal-positive cells was also calculated. (**B**) Gyp-L inhibited cell proliferation. Nuclei of all cells were stained with blue, and nuclei of cells with high DNA replication activities (EdU-positive cells) were stained with red simultaneously. Scale Bar: 100 μm. (**C**) mRNA expression of SASP-related cytokines. The cells were treated with different concentrations of Gyp-L (20, 40, 80 μg/mL) for 24 h and the mRNA expression levels of several cytokines were detected by qRT-PCR. The mRNA expression was normalized by housekeeping gene GAPDH. The students’ two-tailed t test was used for all statistical analysis, with the level of significance set at (***) *p* < 0.005, (**) *p* < 0.01, and (*) *p* < 0.05 vs. control group.

To test whether Gyp-L induces senescence in human cancer cells, human hepatic cancer cell HepG2 and esophagus cancer cell ECA-109 were treated with Gyp-L for 24 h. The senescence associated β-galactosidase (SA-β-gal) staining assay was performed and cells positive for β-galactosidase have the potential for senescence. Indeed, treatment with Gyp-L significantly increased the percentages of SA-β-gal-positive cells (Figure 1A). We then analyzed cell proliferation by 5-ethynyl-2’-deoxyuridine (EdU) staining assay to confirm the impaired proliferation activity of senescent cells. The nuclei of all cells were stained with blue and the nuclei of cells with high DNA replication activities (EdU-positive cells) were stained with red simultaneously. As shown in Figure 1B, the proportion of EdU-positive cells was higher in the control group, which was remarkably reduced in a concentration-dependent manner in the presence of Gyp-L, indicating that Gyp-L inhibited the proliferative activity of cancer cells. Furthermore, we detected the senescence-associated expression of SASP, such as IL-1α, IL-6, TIMP-1, CXCL-1 and CXCL-2, by qRT-PCR (Figure 1C). As expected, the mRNA expression levels of all cytokines were increased by Gyp-L. Together, these results indicate that Gyp-L induces senescence in human cancer cells.

### 2.2. Gyp-L Causes Cell Cycle Arrest

**Figure 2:**
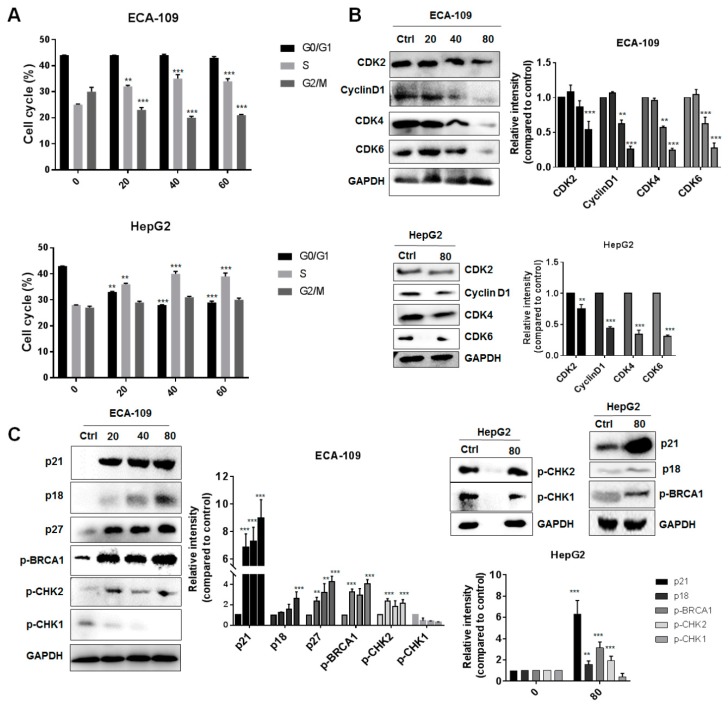
Gyp-L upregulated cell cycle inhibitors. (**A**) Gyp-L causes cell cycle arrest at S phase. The cells were treated with indicated concentrations of Gyp-L for 24 h and cell cycle distribution was analyzed by FACS assay. (**B**,**C**) The cells were treated with Gyp-L for 24 h and cell lysates were subjected to western blot for indicated proteins, including cell cycle kinases and their inhibitor proteins. Densitometric analysis for all western blot bands was shown. GAPDH served as a loading control. The students’ two-tailed t test was used for all statistical analysis, with the level of significance set at (***) *p* < 0.005, (**) *p* < 0.01, and (*) *p* < 0.05 vs. control group.

As cell cycle arrest is another representative characteristic of senescence, we therefore examined cell cycle distribution of cancer cells under Gyp-L treatment. Flow cytometry assay results demonstrated that a progressive increase of cells, retardant in S-phase, occurred in hepatic and esophagus cancer cells when treated with different concentrations of Gyp-L (Figure 2A). Next, we detected the protein levels of several cell cycle kinases (CDKs) that are critical for cell cycle progression. Gyp-L significantly reduced the expression of all cell cycle regulators, such as CDK2, CDK4, CDK6, and cyclin D1, which was consistent with the arrested cell cycle (Figure 2B). Additionally, we evaluated the upstream regulators of CDKs. Two critical signaling pathways, ATM-CHK2-p53 and ATR-CHEK1, are mainly responsible for cell cycle arrest, by activating CDK inhibitor proteins (CKIs), such as p21, to inhibit the activity of CDKs. We found that several CKIs, including p21, p18, and p27 were largely upregulated by Gyp-L (Figure 2C). Besides, we showed that Gyp-L activated cell check kinase CHK2, instead of CHK1, to inhibit cell cycle kinases and cause cell cycle arrest. Finally, BRCA1, the downstream mediator of CHK2 that activates several DNA repairing proteins and cell cycle regulators, such as p53, Rb and PLK1, has also been activated under the treatment of Gyp-L. These results further strengthen the involvement of ATM-CHK2 pathway in controlling cell cycle arrest.

### 2.3. Gyp-L Induces Senescence Via MAPK Signals

**Figure 3:**
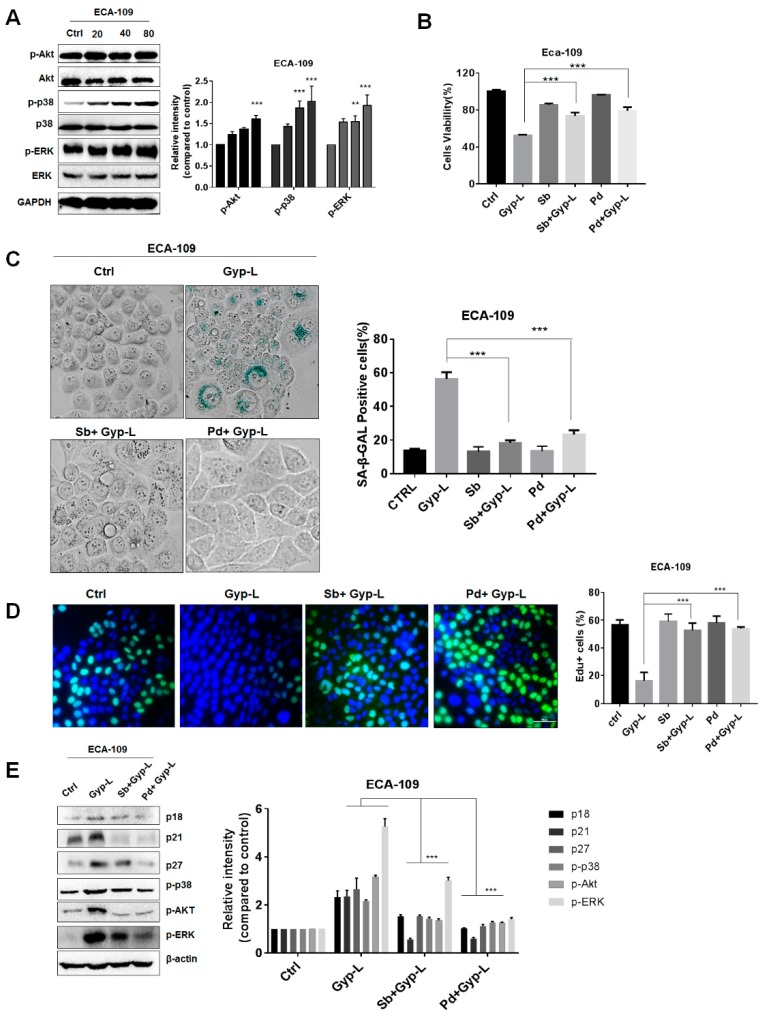
Gyp-L activated MAPK pathways in esophageal cancer cells. (**A**) Gyp-L activated p38 and ERK pathway. GAPDH served as a loading control. (**B**) ECA-109 cells were treated with Gyp-L (80 μg/mL) in the presence or absence of SB203580 (10 μM) or PD98059 (10 μM) for 24 h and cell viability was analyzed by CCK8 assay. SB203580 (10 μM) or PD98059 (10 μM) also inhibited SA-β-gal activity (**C**) and increased cell proliferation (**D**). Nuclei of cells with high DNA replication activities (EdU-positive cells) were stained with green. (E) Chemical inhibitors reduced the protein levels of cell cycle regulators, such as p21, p27 and p18, as well as phosphorylated p38 and ERK in ECA-109 cells. β-actin served as a loading control. Sb: SB203580; Pd: PD98059. The students’ two-tailed t test was used for all statistical analysis, with the level of significance set at (***) *p* < 0.005, (**) *p* < 0.01, and (*) *p* < 0.05.

Next we investigated the possible mechanism involved in Gyp-L-induced senescence. Several intracellular signals, such as MAPK, autophagy, and reactive oxygen species (ROS), have been demonstrated to cause cell cycle arrest and induce senescence. Firstly, we found that Gyp-L activated MAPK signals, mainly through p38 and ERK signaling pathways, in a dose-dependent manner in esophageal cancer (Figure 3A). However, no activation was detected in JNK signaling pathway (date not shown). Inhibition of p38 by specific chemical inhibitor SB203580, or the inhibition of ERK by its upstream kinase inhibitor PD98059, apparently restored cell viability reduced by Gyp-L (Figure 3B). SA-β-gal staining and EdU staining assay clearly demonstrated that single administration of SB203580 or PD98059 had no effect on SA-β-gal activity and cell proliferation. However, combinatory treatment with Gyp-L and SB203580 or PD98059 significantly recovered Gyp-L-induced cellular senescence, and cell proliferation, respectively (Figure 3C,D). In addition, the treatment of inhibitors considerably inhibited the expression of several regulators of cell cycle arrest, including p21, p18, and p27, further confirming the critical role of MAPK signals in Gyp-L-mediated senescence (Figure 3E).

**Figure 4:**
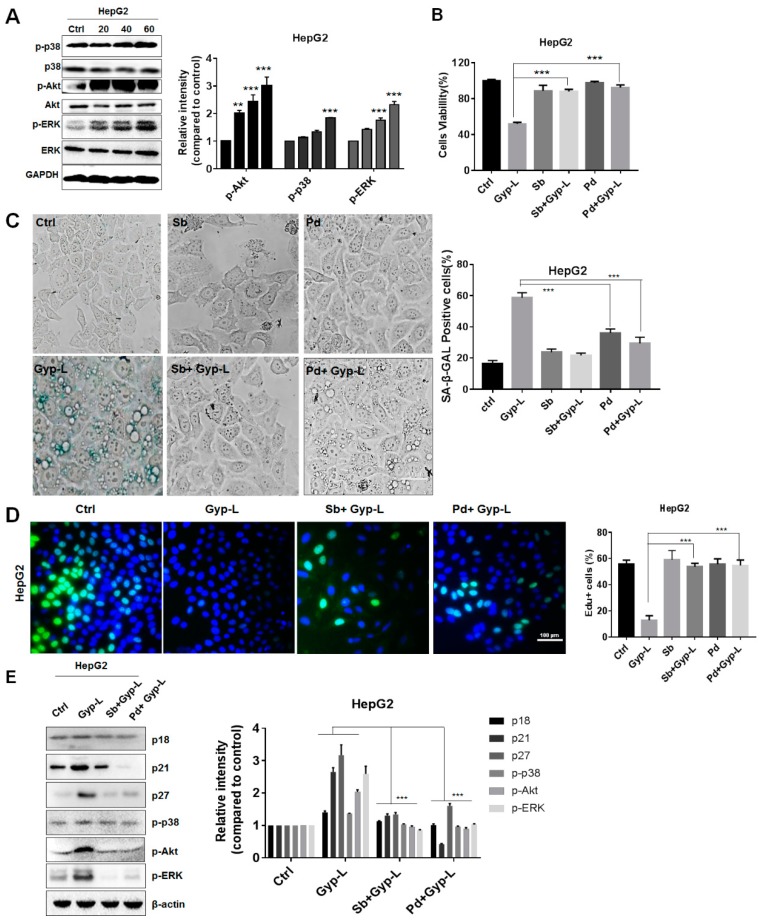
Gyp-L activated MAPK pathways in liver cancer cells. (**A**) Gyp-L activated p38 and ERK pathway in HepG2 cells. GAPDH served as a loading control. (**B**) HepG2 cells were treated with Gyp-L (80 μg/mL) in the presence or absence of SB203580 (Sb, 10 μM) or PD98059 (Pd, 10 μM) for 24 h and cell viability was analyzed by CCK8 assay. SB203580 (10 μM) or PD98059 (10 μM) also inhibited SA-β-gal activity (**C**) and increased cell proliferation (**D**). Nuclei of cells with high DNA replication activities (EdU-positive cells) were stained with green. (**E**) Inhibitors reduced the protein levels of p21, p27, and p18, as well as activating the p38 and ERK pathways in HepG2 cells. The cells were treated with Gyp-L (80 μg/mL) in the presence of Sb (10 μM) or Pd (10 μM) for 24 h and cell lysates were subjected to western blot for the analysis of target proteins. β-actin served as a loading control. The students’ two-tailed t test was used for all statistical analysis, with the level of significance set at (***) *p* < 0.005, (**) *p* < 0.01, and (*) *p* < 0.05.

Furthermore, we tested whether MAPK signals similarly played an important role in Gyp-L-induced senescence in liver cancer cells. As shown in Figure 4A,B, Gyp-L also activated p38 and ERK in a dose-dependent manner in HepG2 cells. Consistently, their inhibition by SB203580 and PD98059 resumed the cell proliferation, impaired by Gyp-L. In addition, treatment of SB203580 or PD98059 alone did not affect cellular senescence, whereas combinatory treatment of SB203580 and PD98059 reduced the ratio of SA-β-gal-positive cells, and increased the ratio of Edu-positive cells, respectively (Figure 4C,D). Finally, the protein levels of p21, p18, and p27 were largely reduced in the simultaneous presence of Gyp-L and inhibitors in HepG2 cells (Figure 4E). Together, these results suggest that p38 and ERK signaling pathways participate in Gyp-L-induced cellular senescence in both esophageal and liver cancer.

### 2.4. Gyp-L Induces Senescence Via NF-Κb Activation

**Figure 5:**
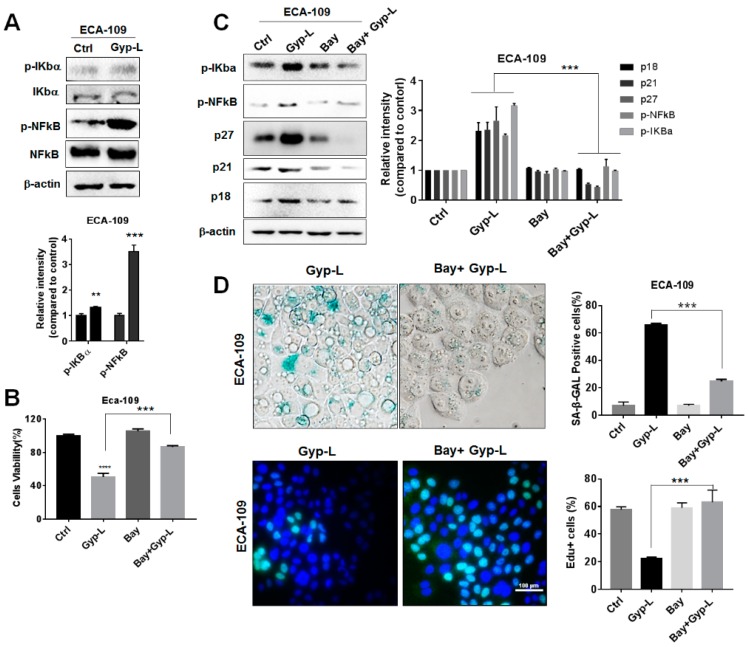
Gyp-L activated NF-κB pathway in esophageal cancer cells. (**A**) ECA-109 cells were treated with Gyp-L (80 μg/mL) for 24 h. β-actin served as a loading control. (**B**) ECA-109 cells were treated with Gyp-L (80 μg/mL) in the presence or absence of Bay11-7082 (Bay, 10 μM) for 24 h and cell viability was analyzed by CCK8 assay. (**C**) Bay also inhibited activated NF-κB and cell cycle regulator which was upregulated by Gyp-L. β-actin served as a loading control. (**D**) Bay significantly inhibited SA-β-gal activity and increased cell proliferation. The students’ two-tailed t test was used for all statistical analysis, with the level of significance set at (***) *p* < 0.005, (**) *p* < 0.01, and (*) *p* < 0.05 vs. Gyp-L-treated group.

NF-κB signaling pathway is another important regulator of cellular senescence. To explore its potential regulation in Gyp-L-induced senescence, a western blotting experiment was performed, which showed that the phosphorylation and activation of NF-κB was markedly up-regulated by Gyp-L in ECA-109 cells (Figure 5A). Treatment with NF-κB inhibitor Bay11-7082 significantly decreased the inhibitory effect of Gyp-L on cancer cell growth (Figure 5B), as well as the percentage of SA-β-gal positive cell population (Figure 5D). Consistent with the SA-β-gal staining, EdU staining of the combinatory treatment of Gyp-L and Bay11-7082 exhibited a remarkable increment of the percentage of proliferating cells (Figure 5D), while single treatment with Bay had no effect. Finally, the majority of cell cycle regulators, such as p21, p27, and p18, were critically reduced by Bay11-7082 (Figure 5C). All these results clearly suggest that NF-κB signal is important for Gyp-L-induced senescence.

**Figure 6:**
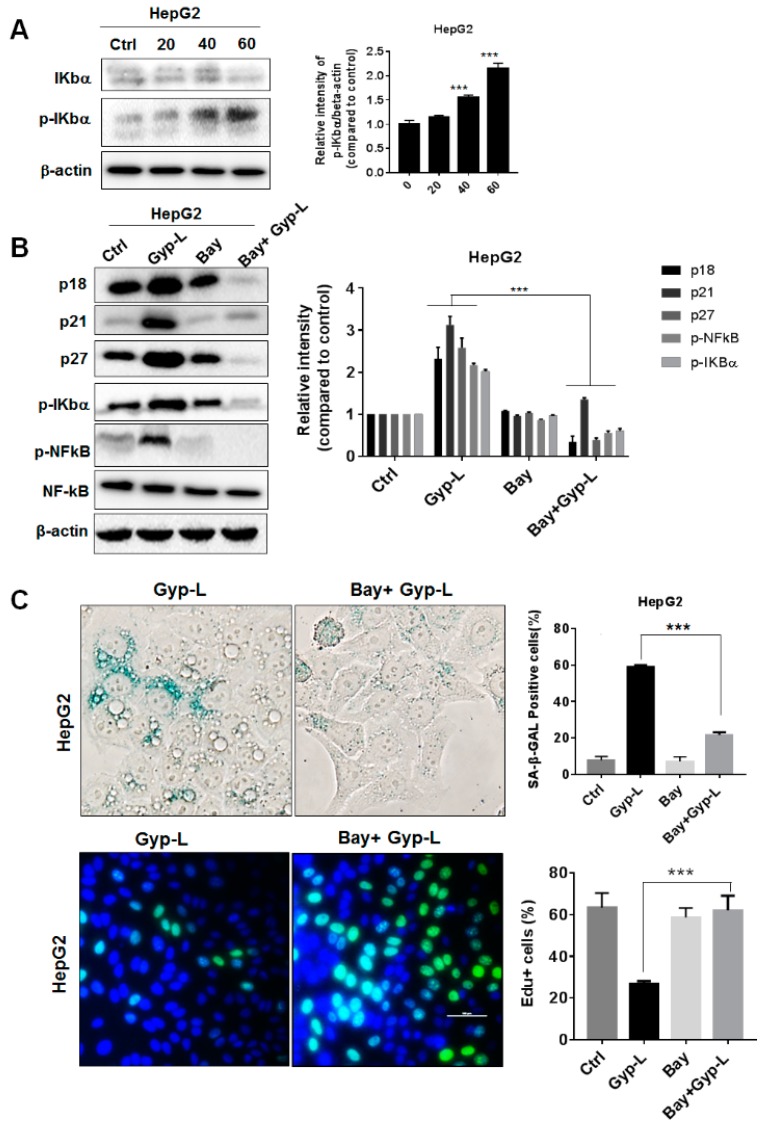
Gyp-L activated NF-κB pathway in liver cancer cells. (**A**) HepG2 cells were treated with different concentrations of Gyp-L for 24 h. The students’ two-tailed t test was used for all statistical analysis, with the level of significance set at (***) *p* < 0.005, (**) *p* < 0.01, and (*) *p* < 0.05 vs. control group. (**B**) HepG2 cells were treated with Gyp-L (80 μg/mL) in the presence or absence of Bay11-7082 (Bay, 10 μM) for 24 h and cell lysates were subjected to western blot. β-actin served as a loading control. (**C**) HepG2 cells treated with Gyp-L and Bay were stained for SA-β-gal or EdU. (***) *p* < 0.005, (**) *p* < 0.01, and (*) *p* < 0.05 vs. Gyp-L-treated group.

In addition, we validated the function of the NF-κB signaling pathway in liver cancer cells. Similar to esophageal cancer, Gyp-L activated NF-κB in a concentration-dependent manner (Figure 6A). The combination treatment of Gyp-L and Bay11-7082 significantly decreased the percentage of SA-β-gal positive cells and the expression of cell cycle regulators (Figure 6B,C). Bay11-7082 also increased the EdU-positive cells (Figure 6C). Therefore, NF-κB signal participated in cellular senescence, caused by Gyp-L.

### 2.5. Gyp-L Enhances the Sensitivity of Cancer Cells Toward Chemotherapy

**Figure 7:**
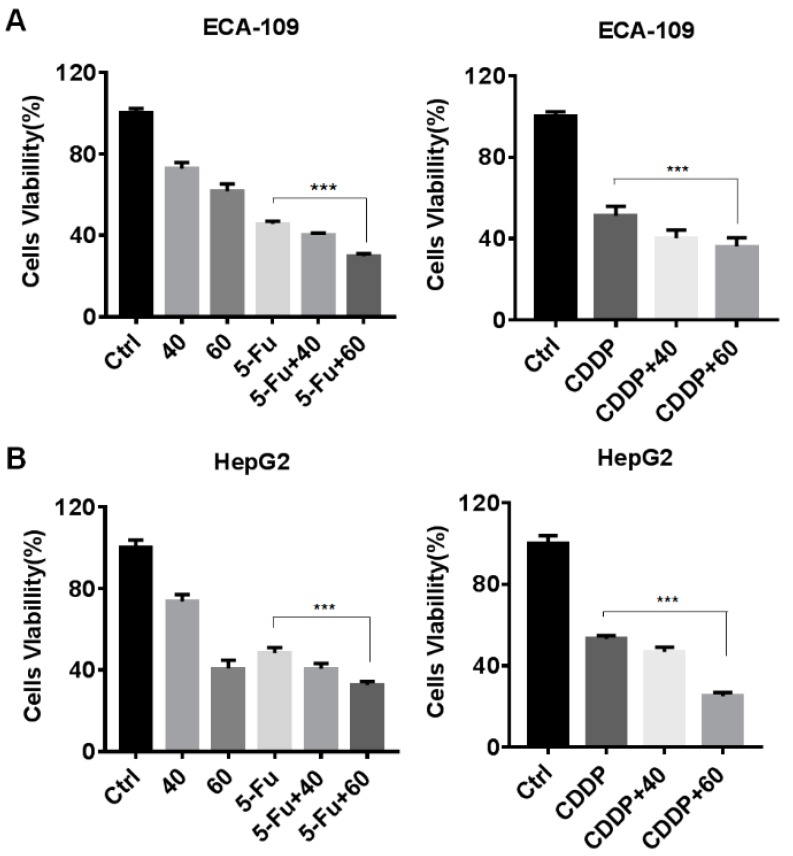
Gyp-L enhanced the cytotoxicity of 5-Fu and CDDP. ECA-109 cells (**A**) and HepG2 cells (**B**) were cotreated with different concentrations of Gyp-L (40 or 60 μg/mL) and 5-Fu (30 μM) or CDDP (10 μM) for 48 h, respectively. Cell viability was analyzed using CCK8. The students’ two-tailed t test was used for all statistical analysis, with the level of significance set at (***) *p* < 0.005, (**) *p* < 0.01, and (*) *p* < 0.05.

To verify whether the combined usage of Gyp-L and the first-line clinical medication, such as cisplatin (CDDP) or 5-fluorouracil (5-Fu), exerted a superior cytotoxic effect on liver and esophageal cancer cells, we treated HepG2 and ECA-109 cells, with cisplatin (10 μM) or 5-fluorouracil (30 μM), for 48 h in the presence or absence of Gyp-L. CCK8 assay revealed that Gyp-L rendered cancer cells more sensitive toward CDDP and 5-Fu (Figure 7A,B). The combined usage exhibited a higher ratio of cell death in both HepG2 and ECA-109 cells, suggesting that Gyp-L is an alternative strategy to overcoming chemoresistance of cancer cells.

### 2.6. Discussion

Natural products are potential resources for anti-cancer agents [23], and increasing evidence has shown the interaction between senescence and natural products. For instance, ganoderiol F, a tetracyclic triterpene isolated from Ganoderma amboinense, induces senescence after 18 days of continuous treatment of HepG2 cells [24]. Resveratrol has been reported to produce senescence in lung cancer cells after 10 to 12 days incubation [25]. Argentatin B, a cycloartane-type triterpene, derived from Parthenium argentatum Gray (guayule), induces senescence in prostate and colon cancer cells [26]. Interestingly, commonly used chemotherapeutic drugs, such as cisplatin, doxorubicin, etoposide, and other topoisomerase inhibitors are capable of inducing senescence of cancer cells, when they are used in very low concentrations [27]. Similarly, we showed here that saponin Gyp-L can inhibit the growth of human liver and esophageal cancer cells by inducing senescence. Gyp-L increased SA-β-gal activity, caused cell cycle arrest at S phase and promoted the secretion of SASP.

Our works revealed that the activation of CDK inhibitor proteins, including p21, p27, and p18, contributed to the senescence and cell cycle arrest triggered by Gyp-L (Figure 2C). Moreover, we demonstrated that Gyp-L induced cellular senescence via the activation of MAPK and NF-κB pathways. Multiple studies have revealed a major role of the p38 and ERK pathway in oncogenic induced senescence, whereas NF-κB pathway is critical for SASP production [28,29]. A likely intermediate between ERK and p38 is reactive oxygen species (ROS), which is induced by oncogene ras and mediates p38 activation [30,31,32]. Indeed, our previous works also clearly demonstrated that Gyp-L was able to trigger ROS [20,21]. It is therefore possible that Gyp-L triggered ROS to activate both p38 and ERK MAPK signaling pathways. Whether a crosstalk exists between MAPK and NF-κB pathway remains to be clarified.

Furthermore, we showed that Gyp-L enhanced the cytotoxicity of chemotherapeutic agent cisplatin and 5-Fu. Our previous works have demonstrated that 5-Fu activated protective autophagy to obtain chemoresistance, whereas inhibiting autophagy by ginsenoside Ro potentiates 5-Fu cytotoxicity via delaying CHK1 degradation and downregulating DNA replication process, resulted in the delayed of DNA repair and the accumulation of DNA damage [33]. Similarly, protective autophagy has been extensively demonstrated to be an important factor for the resistance of cancer cells towards cisplatin [34,35,36]. Considering that Gyp-L possessed the ability to inhibit autophagy [20,21,22], it is reasonable to infer that Gyp-L might render cancer cells more sensitive to cisplatin and 5-Fu by impairing the essential function of autophagy. In our current works, we provided another possibility that Gyp-L activated senescence, especially long-term cell cycle arrest, to enhance the cytotoxicity of 5-Fu and cisplatin. The retarded cell cycle might negatively affect DNA damage repair, and as a result, cell death. Together, these works suggested that Gyp-L provided an optimism for overcoming chemoresistance.

## 3. Materials and Methods

### 3.1. Cell Lines and Culture

HepG2 and ECA-109 cell lines were obtained from the American Type Culture Collection (Rockville, MD, USA). The cell lines were grown in RPMI 1640 medium (Gibco, Grand Island, NY, USA) with 10% fetal bovine serum (FBS, 10100145, Gibco).

### 3.2. Antibodies and Inhibitors

Anti-Mouse secondary antibody (7076), CDK2 (2546), CDK4 (12790), CDK6 (3136), CyclinD1 (2978), Akt (4691), p-Akt (4060), p-CHK1 (2348), p-CHK2 (2197), p-BCA1 (9009), Anti-Rabbit (7074), Iκbα (4814), p-Iκbα (2859), p-JNK (4668), p-p65 (3033), p65 (8242), p18(2896), p21 (2947), p27 (3686), p38 (8690), p-p38 (4511), GAPDH (5174), β-Actin (4970), p-ERK (9101) and ERK (4695) were purchased from Cell Signaling Technology (Danvers, MA, USA). Chemical inhibitors SB203580 (S1076), PD98059 (S1177), Bay11-7082 (S2913) were from Selleck (Shanghai, China). Chemotherapeutic agents 5-Fu (S1209) and cisplatin (s1166) were from Selleck.

### 3.3. Western Blotting

Cells were treated with Gyp-L for 24 h in the presence or absence of inhibitors. Cell lysates were collected and lysed in radioimmunoprecipitation assay (RIPA) buffer (Beyotime, P0013B, Nanjing, China) containing phosphatase inhibitor cocktail (Sigma-Aldrich, P8340) and 1 mM phenylmethylsulfonyl fluoride (PMSF). Total proteins were quantified to the same concentration and were separated by 8-12% SDS-PAGE. After being transferred to the polyvinylidene fluoride (PVDF) membrane, the proteins were blocked with 5% Skim milk and detected with the specific primary antibodies. We used Clarity Western ECL Substrate (BIO-RAD, 1705061, Hercules, CA, USA) with horseradish peroxidase (HRP)-conjugated secondary antibody for imaging. Image acquisition and analysis software was used to quantify band intensity. For densitometric analysis, the ratio of target protein/loading control (GAPDH or β-actin) has been calculated and the ratio of control group was set to 1.

### 3.4. Senescence-Associated β-Galactosidase (SA-β-gal) Staining

The detection of SA-β-gal Activity at pH 6.0 was performed according to the manufacturer’s instructions (C0602, Beyotime, Shanghai, China). The cells were fixed with the fixative solution and stained with the staining solution obtained from the kit. A microscope was used to observe and photograph the stained cells. Total cell numbers and the stained cell numbers were counted randomly from 4 to 9 fields per well. SA-gal-positive cells were calculated as the percentage of positive cells per unit area.

### 3.5. Cell Counting Kit-8 (CCK8) Assay

Cells were seeded in 96-well plate at density of 3 × 103 cells per well for CCK8 Assay (CK04, Dojindo Laboratories, Kumamoto, Japan). Cells were incubated overnight and treated with various concentrations of Gyp-L for 24 h. Then, 10 μL of CCK8 solution was added to each well and incubated at 37 °C for 1 h. The optical density (OD) was recorded at 450 nm.

### 3.6. EdU Staining

Propagation of cells was cytochemically detected according to the manufacturer’s instructions (C0071L, Beyotime, China). Briefly, cancer cells were incubated with the EdU staining buffer for 2.5 h, fixed by 4% polyformaldehyde and stained the nuclear with Hoechst. The stained cells were scanned and photographed under microscope. Furthermore, integrated optical density (IOD) was used to assess EdU-positive cells with Cytation5 (BioTek, Winooski, VI, USA).

### 3.7. FACS Analysis

The cells were fixed in 70% ethanol at 4 °C overnight and then stained with Cell Cycle Assay Kit (C1052, Beyotime, China) for 10 min. All the cells were analyzed by flow cytometry (BD FACSCalibur, Franklin Lakes, NJ, USA).

### 3.8. Quantitative Real-Time PCR (qRT-PCR)

Total RNA extracted with TRIzol was used for reverse transcription, according to the manufacturer’s instructions (Thermo Fisher Scientific, Waltham, MA, USA). qRT-PCR was performed using FastStart Universal SYBR^®^Green Master Mix (ROX) (4913850001, Roche, Basel, Switzerland). The primers sequences were as follows: GAPDH, 5′-GGGAAACTGTGGCGTGAT-3′ (forward) and 5′-GAGTGGGTGTCGCTGTTGA-3′ (reverse); IL-1α, 5′-AGGCTGCAT GGATCAATCTGTGTC-3′ (forward) and 5′-CTTCCTCTGAGTCATTGGCGATGG-3′ (reverse); IL-6, 5′-GGTGTTGCCTGCTGCCTTCC-3′ (forward) and 5′-GTTC TGAAGAGGTGAGTGGCTGTC-3′ (reverse); CXCL-1, 5′-TCCTGCGAGT GGCACTGCTG-3′ (forward) and 5′-CTGGCAGCGCAGTTCAGTGG-3′ (reverse); CXCL-2, 5′-CTGCCAGTGCTTGCAGACC-3′ (forward) and 5′-CTTAACCATG GGCGATGCGG-3′ (reverse); TIMP-1, 5′-CCTGGCTTCTGGCATCCTGTTG-3′ (forward) and 5′-CGCTGGTATAAGGTGGTCTGGTTG-3′ (reverse). The mRNA expression normalization was internal controlled by housekeeping gene GAPDH.

### 3.9. Statistical Analysis

Data shown in this study were representative or the statistics [mean value ± standard deviation (SD)] of the results from at least three independent experiments. The students’ two-tailed t test was used for all statistical analysis, with the level of significance set at (***) *p* < 0.005, (**) *p* < 0.01, and (*) *p* < 0.05. All the statistical analyses were correlated by GraphPad Prism 7 software.

## 4. Conclusions

In summary, the present study demonstrated that Gyp-L induces cellular senescence in human hepatocarcinoma and esophageal cancer cells, which may correlate with the activation of MAPK and NF-κB pathways. Further works should investigate the senescence-inducing effect of Gyp-L in vivo. It is also worth chemically modifying Gyp-L to exhibit greater efficacy, metabolic stability and to provide clinically acceptable pharmacokinetic and pharmacodynamic profiles to humans.
